# Carcinogenesis trajectories

**DOI:** 10.3389/fcell.2025.1671115

**Published:** 2025-12-09

**Authors:** Rui Wang, Zhaopeng Yan

**Affiliations:** 1 Department of Critical Care Medicine, Shengjing Hospital of China Medical University, Shenyang, China; 2 Department of General Surgery, Shengjing Hospital of China Medical University, Shenyang, China

**Keywords:** cancer origin, tumorigenesis, oncogenesis, carcinogenesis, malignant transformation, cancer initiation, tumorigenic pathway

## Abstract

**Introduction:**

Cancer origin patterns significantly influence cancer phenotypes and hallmark characteristics.

**Method:**

In the present review, four distinct carcinogenesis trajectories that contribute to malignant transformation: mutator phenotype, chromosomal instability, dysmetabolism, and stemness, are explored.

**Results and Discussion:**

In the mutator phenotype trajectory, deficiencies in DNA repair or synthesis systems lead to hypermutation and accumulation of oncogenic alterations. The chromosomal instability trajectory involves aneuploidy-induced copy number alterations in oncogenes and tumor suppressor genes. Dysmetabolic carcinogenesis is driven by the accumulation of oncometabolites due to alterations in metabolic genes. The stemness trajectory refers to the malignant transformation of cells possessing stem-like properties under oncogenic stimuli. Each trajectory independently promotes carcinogenesis and endows cancer cells with distinct characteristics. Notably, the primary oncogenic drivers in each trajectory can self-reinforce and form spontaneous-reinforcing loops that amplify oncogenic signals. Although crosstalk exists among trajectories, evidence suggests they are mutually exclusive during cancer origin. Therefore, targeting specific carcinogenesis trajectories and disrupting the self-reinforcing oncogenic loops may represent novel therapeutic strategies. Understanding carcinogenesis trajectories provides a framework for future cancer research and treatment approaches.

## 1 Introduction

Cancer is a group of diseases characterized by the uncontrolled proliferation of transformed cells, which are subject to evolution by natural selection (Brown et al., 2023). Challenges in cancer management are largely attributed to the inherent complexities of cancer biology (Hanahan and Weinberg, 2011; Hanahan, 2022), as evidenced by its extensive hallmarks and intricate crosstalk. Although the hallmarks of cancer are universal features of malignant cells (Hanahan, 2022), their significance varies across different cancer types and context. For example, certain cancers exhibit hypermutations, whereas others may present somatic copy number variants. The variable significance of cancer hallmarks can be attributed to tumor heterogeneity, plasticity, and origin variations (Jassim et al., 2023). The pattern of cancer origin is particularly crucial in determining cancer phenotypes. In this article, we aim to explore and delineate several trajectories through which carcinogenesis occurs, providing a comprehensive overview of the mechanisms underlying cancer initiation. We refer to these pathways as “carcinogenesis trajectories.” This article solely focuses on the process of cancer origin, rather than cancer progression.

Typically, there are four primary carcinogenic trajectories: mutator phenotype, chromosomal instability (CIN), dysmetabolism, and stemness. The mutator phenotype trajectory posits that deficiencies in DNA repair or synthesis systems exert a mutator effect. Specifically, initially occurring mismatch repair deficiency can impair the function of other normal mismatch repair genes, forming a “mutator-mutates-other-mutators” loop. This loop progressively damages DNA repair systems and drives carcinogenesis by accelerating mutation rates and accumulating oncogenic mutations. The chromosomal instability (CIN) trajectory suggests that CIN induces aneuploidy, which in turn reduces chromosomal stability and exacerbates aneuploidy—forming a “CIN-aneuploidy-CIN” loop. This loop leads to progressive copy number alterations (CNAs) in genes; once CNAs in cancer-related genes reach the threshold for malignant transformation, cancer originates. The dysmetabolic trajectory proposes that the accumulation of oncometabolites (a consequence of alterations in metabolism-related genes) amplifies dysmetabolism, establishing a “dysmetabolism-oncometabolite-dysmetabolism” loop. These oncometabolites then promote oncogenic signaling and induce cancer origin. The stemness trajectory indicates that cells with stemness properties undergo malignant transformation in response to oncogenic stimuli, generating cancer cells that retain stemness characteristics. In the carcinogenesis process, oncogenic drivers can self-maintain and amplify, triggering an oncogenic cascade that accumulates oncogenic signals until cellular transformation occurs. Notably, the longer the carcinogenic loop persists, the more oncogenic signals are activated—and consequently, the higher the probability of malignant transformation. Therefore, targeting these carcinogenic trajectories and disrupting self-reinforcing oncogenic loops may present a potential therapeutic strategy.

## 2 Mutator phenotype carcinogenesis trajectory

### 2.1 Mutator phenotype theory

Accumulation of oncogenic mutations is a predominant mechanism underlying carcinogenesis. Various genotoxic agents, such as ionizing radiation, ultraviolet light from sunlight, and chemicals, can result in DNA damage, accumulation of mutations, and malignant transformation (Ciccia and Elledge, 2010). Under normal conditions, DNA damage repair systems maintain a low mutation rate in normal cells, which is typically insufficient to accumulate the numerous oncogenic mutations required for malignant transformation (Fox et al., 2013; Loeb, 2016). Therefore, the existence of “mutation accelerators” has been proposed to account for the carcinogenesis process (Loeb, 2010; Kentsis and Frank, 2020). In this context, the mutator phenotype theory has been postulated, suggesting the presence of a specific phenotype that contributes to the acceleration of the mutation rate and malignant transformation. In general, the mutator phenotype is characterized by deficiencies in mismatch repair genes (Bak et al., 2014; Roberts and Gordenin, 2014) and polymerase proofreading mechanisms (Albertson et al., 2009), which can lead to hypermutation (Shlien et al., 2015) and increase susceptibility to various cancers (Jeggo et al., 2016).

### 2.2 Mismatch repair deficiency and microsatellite instability

Mismatch repair genes, including *MLH1*, *MSH2*, *MSH6*, and *PMS2*, correct spontaneous mutations in repetitive DNA sequences (Pećina-Šlaus et al., 2020; Vilar and Gruber, 2010). The predominant consequence of a deficient mismatch repair system is microsatellite instability (MSI), which is characterized by length alterations in simple repeated microsatellite sequences and the accumulation of frameshift mutations in repeated sequences of target genes. Various mechanisms can compromise mismatch repair function and lead to MSI. First, MSI can be caused by direct germline or somatic mutations in mismatch repair genes. Second, epigenetic mechanisms can cause MSI. For example, the 3′end deletion of the epithelial cell adhesion molecule gene, which is located upstream of *MSH2*, can lead to the methylation of the *MSH2* promoter and subsequently cause MSI (Ligtenberg et al., 2009). Epigenetic silencing of the *MLH1* promoter via CpG island hypermethylation can also lead to MSI (Herman et al., 1998; Leung et al., 1999). Third, in some scenarios, the MSI phenotype can emerge without genetic or epigenetic mismatch repair gene alterations. For instance, ARID1A facilitates the recruitment of MSH2 to chromatin, enabling effective mismatch repair during DNA replication. Inactivation of ARID1A impairs the mismatch repair effect of MSH2 and can lead to an MSI phenotype (Shen et al., 2018). Moreover, trimethylation of histone H3 at Lys36 (H3K36 me3) is an epigenetic histone mark required for the mismatch repair process (Li et al., 2013). Loss of the H3K36 trimethyl-transferase SETD2 impairs the mismatch repair process and induces an MSI phenotype (Li et al., 2013). Fourth, overexpression of miR-155 or miR-21 can downregulate the expression of mismatch repair proteins, thereby inducing an MSI phenotype (Valeri et al., 2010a; Valeri et al., 2010b).

MSI occurs in various tumors (Quaas et al., 2021; Hause et al., 2016), serving as a hallmark of cancer. However, MSI also drives carcinogenesis, primarily by promoting frameshift mutations (Yamamoto and Imai, 2015; Imai and Yamamoto, 2008) that affect various target genes. These include DNA repair genes, such as *MSH3* and *MSH6*. Mutations in these genes can further increase mutation rates, exacerbate MSI, and predispose to carcinogenesis (Malkhosyan et al., 1996). Cancer-related genes are also direct targets of frameshift mutations by MSI; the activation of oncogenes and inactivation of tumor suppressor genes confer growth and survival advantages that promote carcinogenesis (Gylfe et al., 2013; Tuupanen et al., 2014; An et al., 2015). For example, the pro-apoptotic gene BAX can be inactivated by MSI-induced frameshift mutations, contributing to oncogenesis (Woerner et al., 2005). In addition, MSI can target epigenetic modifier genes, such as histone deacetylase 2 (*HDAC2*) (Ropero et al., 2006) and histone methyltransferase genes (Choi et al., 2014). Notably, altered histone modifications resulting from these mutations are closely related to carcinogenesis (Calcagno et al., 2013).

### 2.3 Polymerase proofreading deficiency

DNA polymerases are enzymes involved in DNA synthesis. Heterozygous mutations in DNA polymerases can abrogate their ability to excise mis-incorporated non-complementary nucleotides, leading to a high frequency of single-nucleotide variant mutation rate and the development of a hypermutated phenotype (Liu et al., 2018; Goldsby et al., 2002). Mutations in DNA polymerases can increase susceptibility to various cancers (Rayner et al., 2016; Magrin et al., 2021). The DNA polymerase epsilon (*POLE*) gene encodes the catalytic subunit of DNA polymerase epsilon, whereas the polymerase delta 1 (*POLD1*) gene encodes the catalytic subunit of DNA polymerase delta (Mur et al., 2020). Notably, loss-of-function mutations in *POLE* and *POLD1* can decrease the fidelity of DNA replication, leading to a hypermutation phenotype and ultimately promoting carcinogenesis (Venkatesan et al., 2007).

### 2.4 Hallmarks of mutator phenotype carcinogenesis trajectory

The mutator phenotype carcinogenesis trajectory promotes carcinogenesis through the rapid accumulation of oncogenic genetic or epigenetic alterations via MSI-induced frameshift mutations. Several hallmark characteristics define this carcinogenesis trajectory. First, this trajectory is characterized by a hypermutation phenotype, accompanied by low levels of somatic copy number alterations and chromosomal instability (Camps et al., 2006). Second, it represents an efficient pathway to cancer development. Specifically, while carcinogenesis via the chromosomal instability trajectory typically requires more than 10 years, the mismatch repair deficiency trajectory can lead to cancer within just a few years (De’ Angelis et al., 2018). Third, this trajectory exhibits a high tumor mutational burden and increased production of neoantigens, correlating with enhanced response to immunotherapy (Magrin et al., 2021; Rochefort et al., 2021).

**FIGURE 1:**
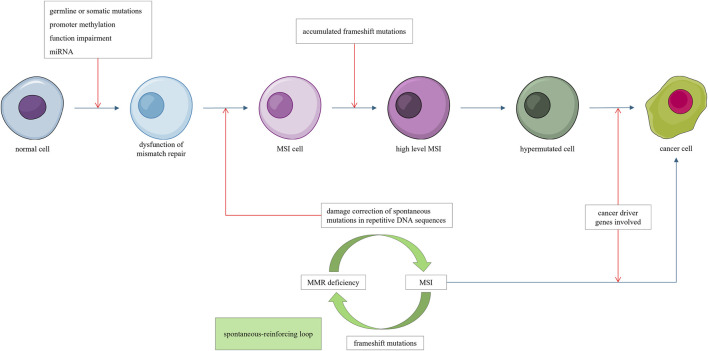
Mismatch repair deficiency leads to microsatellite instability (MSI). Frameshift mutations induced by MSI impair addition mismatch repair genes, forming a self-reinforcing “mutator mutate other mutator” loop, progressively promoting hypermutations. Once a sufficient number of cancer driver genes are affected, cancer emerges.

Notably, MSI is a continuous, rather than discrete phenotype (Hause et al., 2016). Deficiency in mismatch repair genes can lead to MSI, whereas frameshift mutations in *MSH3* and MSH6 can further aggravate mismatch repair deficiency and increase the level of MSI (Malkhosyan et al., 1996), creating a “mutator mutates other mutators” phenotype. This self-reinforcing “mutator–MSI–mutator” loop can progressively alter gene expression through frameshift mutations until sufficient cancer-related genes are affected, leading to cancer initiation (Figure 1).

## 3 Chromosomal instability carcinogenesis trajectory

### 3.1 Chromosomal instability and aneuploidy

Chromosomal instability (CIN) is a type of genomic instability characterized by alterations in chromosome copy number or chromosome structure. DNA damage, particularly in regions adjacent to telomeres, may lead to chromosome fusion and trigger a breakage–fusion–bridge cycle. During this process, dicentric chromosomes form chromosome bridges, which break during subsequent cell divisions, leading to CIN (Murnane, 2012; Chakravarti et al., 2021). Defects in any cellular process involved in DNA replication, chromosomal segregation, and DNA repair (such as homologous recombination deficiency) may potentially lead to CIN. CIN is commonly induced by factors such as ionizing radiation, tobacco smoke constituents (Saletta et al., 2007), viral infections (Cosper et al., 2023; Shumilov et al., 2017), and aging.

Aneuploidy, a characteristic consequence of CIN, refers to copy number alteration affecting either the entire chromosome arm (excluding the short arms of acrocentric chromosomes) or whole chromosomes (Ben-David and Amon, 2020). Aneuploidy represents a manifestation of an imbalanced karyotype (Schukken and Foijer, 2018) and typically arises from errors in chromosomal segregation in euploid cells. Various mechanisms contribute to aneuploidy, including the activation of specific oncogenes (Woo and Poon, 2004), oxidative or mechanical stress (Li and Zhu, 2022), centrosome overduplication (Vitre and Cleveland, 2012), DNA replication stress (Burrell et al., 2013), incorrect kinetochore–microtubule attachments (Li and Zhu, 2022), hypomethylation (Gaudet et al., 2003), and alterations in centromeric DNA and its binding factors (Worrall et al., 2018; Dumont et al., 2020).

### 3.2 Effects of aneuploidy on carcinogenesis

Aneuploidy simultaneously affects a large number of genes and can lead to dramatic alterations in gene dosage, gene expression profiles (Sdeor et al., 2024), cell biology and immune activity (Kuang and Li, 2022). It plays diverse roles in determining cell fate, and its most prominent effect is the modulation of cellular fitness. Generally, aneuploidy induces imbalanced protein production and increased proteotoxic stress, which can reduce cell fitness, disrupt normal cellular biological activity, and lead to cell death (Zhu et al., 2018; Ben-David and Amon, 2020). Notably, aneuploidy can inhibit carcinogenesis through its detrimental and lethal effects on affected cells (Hirpara et al., 2018; Williams et al., 2008). For example, aneuploidy-induced p53 activation can inhibit proliferation and induce apoptosis (Santaguida and Amon, 2015). Sometimes, aneuploidy exhibits a neutral effect because the resulting copy number alterations do not significantly change gene expression profile owing to compensatory or buffering mechanisms, or because the altered expression is neither advantageous nor detrimental for cell survival (Sdeor et al., 2024). Rarely, specific copy number alterations may confer adaptive phenotypes, promote proliferation, and increase cell fitness (Pavelka et al., 2010; Bollen et al., 2021; Ben-David et al., 2014). This survival advantage allows cells to accumulate additional copy number alterations.

Similar to CIN, aneuploidy is not only a hallmark of cancer (Taylor et al., 2018) but also a driver of cancer. However, aneuploidy alone is insufficient to initiate cancer (Kuang and Li, 2022; Sdeor et al., 2024; Vasudevan et al., 2021). The first stage of carcinogenesis involving aneuploidy requires fitness selection, where aneuploid cells that fail to successfully adapt to genomic alterations are eliminated (Sdeor et al., 2024). Notably, low levels of aneuploidy can suppress carcinogenesis (Williams et al., 2008; Stingele et al., 2012), whereas a high degree of aneuploidy promotes it (Taylor et al., 2018; Buccitelli et al., 2017; Davoli et al., 2017). This phenomenon may be explained by the fact that only aneuploid cells with sufficient fitness survive and accumulate extensive aneuploidy.

The second stage of aneuploidy-mediated carcinogenesis involves copy number alterations in cancer-related genes. In aneuploid cells, chromosomal missegregation can lead to copy number gains in oncogenes and loss of heterozygosity in tumor suppressor genes. As alterations in as few as two to eight driver genes can result in cancer (Hart et al., 2015; Alonso-Curbelo et al., 2021), once copy number alterations affect a sufficient number of cancer-related genes and reach a transformation threshold, cancer initiation occurs. For example, in glioblastoma tumor samples, a gain of chromosome 7 and loss of chromosome 10 have been observed (Brennan et al., 2013; Bredel et al., 2009); chromosome 7 carries oncogenes, typically *EGFR* and *HGFR*, while chromosome 10 harbors the tumor suppressor gene *PTEN* (Körber et al., 2019).

### 3.3 Hallmarks of chromosomal instability carcinogenesis trajectory

The chromosomal instability carcinogenesis trajectory refers to tumorigenicity driven by aneuploidy-induced fitness selection and copy number alterations in cancer-related genes (Sack et al., 2018; Davoli et al., 2013). This trajectory is characterized by high somatic copy number alterations, low mutation counts, and the involvement of p53. Functional p53 can be activated by aneuploidy (Santaguida and Amon, 2015) and induce apoptosis in aneuploid cells (Soto et al., 2017; Thompson and Compton, 2010). Given that p53 can reduce the fitness of these cells, loss-of-function mutations in this gene are favored during the CIN carcinogenesis trajectory. This hypothesis is supported by the fact that colorectal cancer cells exhibiting CIN are more likely to acquire p53 mutations at the same time (Guinney et al., 2015).

**FIGURE 2:**
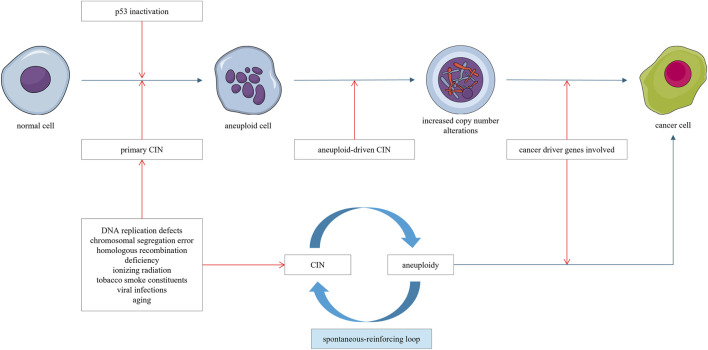
Chromosomal instability (CIN) leads to the emergence of aneuploid cells. Aneuploidy, in turn, can drive genomic instability and aggravate CIN. This establishes a self-reinforcing “CIN–aneuploidy–CIN” loop, characterized by progressively accumulating copy number alterations. Once a sufficient number of cancer-related genes are affected, cancer emerges.

As mentioned above, CIN can cause aneuploidy, which in turn can lead to massive cellular consequences, including further increases in CIN (Chunduri and Storchová, 2019). The degree of CIN depends on the identity and number of aneuploid chromosomes (Zhu et al., 2012). For example, aberrant expression of mitotic transcription regulators induced by aneuploidy can further increase the degree of CIN in breast cancer (Pfister et al., 2018). Aneuploidy exerts complex effects on genomic stability. In certain contexts, it may confer a genome-stabilizing function (Duesberg et al., 1998; Li et al., 2009; Bökenkamp et al., 2025), whereas in other scenarios, aneuploidy itself is sufficient to induce structural instability of chromosomes (Fabarius et al., 2003; Potapova et al., 2013; Lamm et al., 2016). Aneuploidy disrupts the balance of enzyme complexes and alters the dosage of gene products encoded on aneuploid chromosomes. Specifically, dysregulation of genes responsible for DNA synthesis, maintenance, and nucleotide pool homeostasis can trigger subsequent DNA breakage, thereby eliciting chromosomal deletions, amplifications, and rearrangements (Fabarius et al., 2003; Potapova et al., 2013; Lamm et al., 2016). Notably, aneuploidy induces CIN, which in turn amplifies the degree of aneuploidy, creating a spontaneous, self-reinforcing “CIN–aneuploidy–CIN” loop that continuously drives the carcinogenesis process (Figure 2) (Potapova et al., 2013; Fabarius et al., 2003).

## 4 Dysmetabolic carcinogenesis trajectory

### 4.1 Dysregulated metabolisms and carcinogenesis

Cell metabolism can directly control cell survival, proliferation, and differentiation, which are intricately correlated with carcinogenesis (Brunner and Finley, 2023). Dysregulated metabolism is both a hallmark of cancer and an important inducer of its development (Pavlova et al., 2022). For example, fatty acid metabolism, oxidative phosphorylation, and the citric acid cycle are enhanced in the early stages of precancerous lung squamous cell carcinoma lesions (Nie et al., 2021; Mascaux et al., 2019). These data indicate that metabolic remodeling begins early in the precancerous stages and that mutations in metabolic enzymes may act as initial drivers of carcinogenesis. Moreover, mutations in metabolic regulatory genes can lead to aberrant metabolism and predispose individuals to various tumors (Tomlinson et al., 2002; Clark et al., 2014). Environmental factors, such as hypoxia, local nutrient availability, microbiota, chronic inflammation, and diet, can also promote tumor initiation, at least partly through metabolic mechanisms (Brunner and Finley, 2023). Furthermore, cancer transformation can occur without any mutations, driven only by disruptions in cellular bioenergetics (Losman et al., 2013).

### 4.2 Oncometabolites in carcinogenesis

The mechanisms by which metabolic dysregulation leads to carcinogenesis are typically explained by the “oncometabolite theory.” This theory posits that the accumulation of oncometabolites, induced by mutations in mitochondrial DNA or nuclear genes encoding key metabolic enzymes, triggers oncogenic signaling cascades (Sciacovelli and Frezza, 2016). Oncometabolites are small-molecule components that accumulate due to disruptions in the tricarboxylic acid cycle (Krebs cycle). Their accumulation can drive carcinogenesis by promoting oncogenic signaling in the absence of other genetic alterations (Liu and Yang, 2021; Yong et al., 2020; Losman et al., 2013). Some studies describe oncometabolites as “drivers” of carcinogenesis, whereas other studies define oncometabolites as “products of cancer cells” (Yang et al., 2013). In this review, we discuss oncometabolites from the perspective of their role as “drivers” of carcinogenesis.

Typical oncometabolites include fumarate (Yang et al., 2012), succinate, 2-hydroxyglutarate (Du and Hu, 2021), lysophosphatidic acid (Karalis and Poulogiannis, 2024), and lactate (San-Millán et al., 2019; Raychaudhuri et al., 2019; Pandkar et al., 2023). Oncometabolite accumulation can be induced by various factors, including mutations in genes encoding key metabolic enzymes, hypoxia (Intlekofer et al., 2015; Wise et al., 2011), hyperglycemic conditions (Manuel et al., 2020; Blatnik et al., 2008), or mitochondrial dysfunction (Baysal et al., 2000). For example, fumarate hydratase (FH) is a nuclear-encoded Krebs cycle enzyme complex that mediates the reversible reaction between fumarate and malate (Pollard et al., 2005). Mutations in the gene encoding FH result in the accumulation of large amounts of cytoplasmic oncometabolite fumarate (Tomlinson et al., 2002). Hyperglycemia can also induce fumarate accumulation (Manuel et al., 2020) by impairing mitochondrial function (Nishikawa et al., 2000; Thomas et al., 2012). Fumarate accumulation can also be induced by high glucose levels in combination with metformin treatment (Pollard et al., 2005). Moreover, succinate dehydrogenase (SDH) is a Krebs cycle enzyme complex (mitochondrial enzyme) that converts succinate to fumarate to fuel the Krebs cycle. Loss-of-function mutations in SDH-encoding genes disrupt the assembly of respiratory complex II and compromise succinate oxidation, leading to the accumulation of the oncometabolite succinate (Pollard et al., 2005). Another example is isocitrate dehydrogenase 1 and 2 (IDH1 and IDH2, respectively), which are homodimeric enzymes that convert isocitrate to α-ketoglutarate in an NADP + -dependent manner during the Krebs cycle (Xu et al., 2011). Overexpression of genes encoding IDH increases enzyme activity and leads to the accumulation of oncometabolites, including D2-hydroxyglutarate (D2-HG) (Xu et al., 2011; Ward et al., 2010; Dang et al., 2009). The accumulation of 2-HG can also be driven by hypoxia (Wise et al., 2011; Oldham et al., 2015), phosphoglycerate dehydrogenase (Fan et al., 2015), or the MYC pathway (Terunuma et al., 2014).

Oncometabolites can promote oncogenic signaling through multiple mechanisms: (1) Oncometabolites can directly promote oncogenic signaling by regulating the expression of hypoxia inducible factor (HIF) genes (Laukka et al., 2016), which in turn promote oncogenic signals and carcinogenesis (Adam et al., 2011). For example, fumarate can competitively inhibit HIF prolyl hydroxylase, preventing the proteasomal degradation of HIF (Yogev et al., 2010). Moreover, HF can induce pseudohypoxia and promote HIFs (Shanmugasundaram et al., 2014; Sudarshan et al., 2009). Succinate accumulation can lead to aberrant stabilization of HIFs, even under normoxic conditions (Selak et al., 2005). Accumulation of fumarate and 2-HG can activate the mTOR axis, which plays a pro-oncogenic role (Esparza-Moltó and Cuezva, 2018; Sourbier et al., 2014). Moreover, R-2HG accumulation can induce hypersuccinylation in the mitochondria and promote resistance to apoptosis via the BCL-2 pathway (Li et al., 2015). (2) Oncometabolites can promote a CpG island methylation phenotype (CIMP) (Whitehall et al., 2014); promoter hypermethylation associated with CIMP can inactivate tumor suppressor genes, thereby promoting carcinogenesis (Otani et al., 2013). For example, *IDH1* mutations can induce CIMP through the production of oncometabolites 2-HG (Whitehall et al., 2014). Mutations in *SDH* can lead to CIMP by inhibiting 2-OG-dependent histone and DNA demethylases (Letouzé et al., 2013). In addition, succinate accumulation can decrease histone methylation by inhibiting histone lysine demethylases, leading to promoter hypermethylation (Xiao et al., 2012). (3) Oncometabolites can induce excessive production of reactive oxygen species (ROS) (Ishii et al., 2005), whose accumulation can cause irreversible DNA damage and protein oxidation, thereby promoting oncogenic signaling (Ziech et al., 2011; Moreno-Sánchez et al., 2013).

The metabolic-related genes mutations can independently promote carcinogenesis. For example, FH translocates from the cytosol to the nucleus upon DNA damage (Yogev et al., 2010). Mutations in *FH* increase cellular sensitivity to DNA damage induced by ionizing radiation (Johnson et al., 2018). Similarly, *IDH* mutations disrupt chromosomal topology and induce oncogene expression (Flavahan et al., 2016). Notably, these carcinogenesis mechanisms occur independently of the effects of oncometabolites.

### 4.3 Hallmarks of the dysmetabolic carcinogenesis trajectory

**FIGURE 3:**
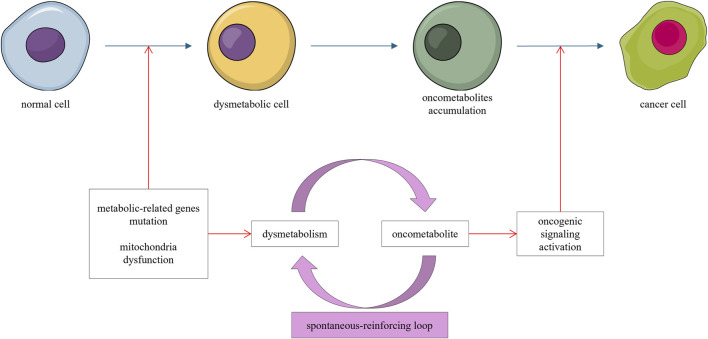
Mutations in metabolism-related genes or mitochondrial dysfunction lead to accumulation of oncometabolites. This accumulation further dysregulates metabolism, forming a self-reinforcing loop that promotes oncogenic signaling and carcinogenesis.

The dysmetabolic carcinogenesis trajectory refers to the aberrant accumulation of oncometabolites resulting from genetic or epigenetic alterations in metabolism-related genes, which promote oncogenic signaling and carcinogenesis. Oncometabolites arising from dysregulated metabolism can further disrupt metabolic processes and promote oncometabolite production (Li et al., 2015), forming a spontaneous, self-reinforcing “dysmetabolism–oncometabolites–dysmetabolism” loop that drives carcinogenesis (Figure 3).

## 5 Stemness carcinogenesis trajectory

The cancer stem cell theory suggests that cancer originates from normal tissue stem cells or stem-like cells. Notably, cells with stem and progenitor characteristics in normal tissues are ideal candidates for transformation owing to their long lifespan, which enables the accumulation of adequate oncogenic mutations (Smalley and Ashworth, 2003), inherent self-renewal and proliferation abilities (Dontu et al., 2003), multidirectional differentiation ability, and access to stemness-maintaining signals from their niches. Malignant transformation has been observed in various stem cells, including neural (Wang et al., 2009), mammary basal (Molyneux et al., 2010), hepatic (Chiba et al., 2010), thyroid (Nagayama et al., 2016), prostate (Losada-García et al., 2023), and hematopoietic stem cells (Shlush et al., 2014). In this section, we briefly review the emergence of stem-like cells and focus on stem cell transformation.

### 5.1 Cells with stemness identity

The stem cell population represents not only distinct cell types but also a plastic phenotype (Thankamony et al., 2020; Raz and Yamashita, 2025). Non-stem cells can acquire stem cell-like properties under certain conditions. First, stem cells depend on their microenvironments, gaining or losing their stemness identity as they enter or exit the stem cell niche (Gehart and Clevers, 2019). For example, the transcription factor Achaete-Scute Homolog 2 can lead to the dedifferentiation of crypt cells, enabling them to acquire stem cell status in the absence of Leucine-Rich Repeat-Containing G-Protein Coupled Receptor 5 (LGR5+) stem cells (Murata et al., 2020). Second, the stemness of normal tissue stem cells is maintained by developmental signaling pathways. Aberrant activation of developmental signals through genetic or epigenetic alterations can induce non-stem cells to adopt a stem-like state. For instance, the activation of NF-κΒ can stimulate WNT signaling, leading to intestinal villus cell dedifferentiation and acquisition of stem cell properties (Schwitalla et al., 2013). Third, genetic epigenetic alterations affecting differentiation-related processes also contribute to stemness acquisition. For example, the Sterile Alpha Motif Domain (SMAD4) is a transcription factor that promotes differentiation. The loss of SMAD4, combined with the activation of the WNT pathway, impairs normal differentiation and drives intestinal epithelial cells toward a stem cell-like phenotype (Perekatt et al., 2018). Therefore, in the absence of pre-existing stem cells, differentiated cells can acquire stemness identity and become potential targets for malignant transformation (Bajaj et al., 2020).

### 5.2 Stem cell transformation

The initial level of stemness, together with secondary oncogenic drivers, determines the likelihood and outcome of cellular transformation. Stemness level influences the transformation potential; generally, undifferentiated stem cells are more responsive to oncogenic mutations than their differentiated counterparts (Lytle et al., 2018). Moreover, oncogenic drivers can transform undifferentiated cells but not differentiated cells. For instance, the activation of Sox2 and Stat3 can promote the malignant transformation of undifferentiated esophageal basal cells but not differentiated suprabasal cells (Liu et al., 2013). In response to *Ras* and *p53* alterations, hair follicle stem cells can transform into squamous carcinoma cells, whereas their progeny, the transit-amplifying cells, cannot form benign tumors under the same genetic conditions (White et al., 2011; Lapouge et al., 2011). Melanoblasts, the precursors of melanocytes, are responsive to oncogenic mutations and undergo malignant transformation more readily than differentiated melanocytes (Baggiolini et al., 2021). Similarly, *APC* deletion leads to transformation in intestinal stem cells but not in short-lived transit-amplifying cells (Barker et al., 2009). Moreover, the level of stemness contributes to the transformed cancer types. For example, BCR–ABL translocation in hematopoietic stem cells leads to chronic myelogenous leukemia, whereas in progenitor cells, it leads to acute lymphoid leukemia (Li et al., 1999; Neering et al., 2007). Loss-of-function mutations in the tumor suppressor genes *P53*, *NF1*, and *PTEN* of neural stem cells, neural progenitors, and oligodendrocyte progenitors induce distinct subtypes of glioblastoma (Alcantara Llaguno et al., 2015). Secondary oncogenic drivers also contribute to malignant transformation and influence the resulting cancer type. For example, activation of hedgehog signaling in either neuronal stem or granule precursor cells can give rise to medulloblastomas with similar molecular phenotypes (Schüller et al., 2008; Yang et al., 2008). Finally, whether a specific signaling acts as a stemness maintainer or a secondary oncogenic driver depends on the cellular context. For example, although Sonic hedgehog signaling generally plays a role in maintaining stemness, its activation in neuronal progenitors leads to medulloblastoma formation (Yang et al., 2008), acting as a secondary oncogenic driver in carcinogenesis.

### 5.3 Hallmarks of stemness carcinogenesis trajectory

**FIGURE 4:**
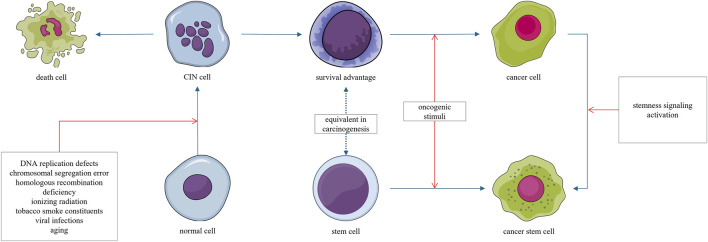
Normal cells can acquire chromosomal instability (CIN) through radiation, chemical carcinogens, or viruses. Most cells exhibiting CIN ultimately die, as CIN is generally disadvantageous to survival. However, a small subset of cells may acquire survival and proliferative advantages through the generation of aneuploidy; this explains the latent period in the carcinogenesis process. Cells that gain such survival and proliferative advantages may undergo malignant transformation upon acquiring even minimal additional oncogenic signals. Stem cells, which inherently possess a long lifespan and intrinsic survival advantages, may undergo malignant transformation under oncogenic stimuli.

The stemness carcinogenesis trajectory refers to the malignant transformation of stem-like cells under secondary oncogenic stimuli. This carcinogenesis trajectory is characterized by a stemness phenotype and high degree of aggressiveness. For example, medulloblastoma originating from stem cells progresses more rapidly than that arising from progenitor cells (Yang et al., 2008). This heightened malignancy may be attributed to the emergence of cancer stem cells (Bajaj et al., 2020; Smith et al., 2015; Zhang et al., 2008), which are associated with disease progression, metastasis, recurrence, and resistance to treatment (Chu et al., 2024). The stemness trajectory is closely associated with, and may be considered as a shortcut of, the chromosomal instability trajectory (Figure 4).

## 6 Crosstalk among carcinogenesis trajectories

**FIGURE 5:**
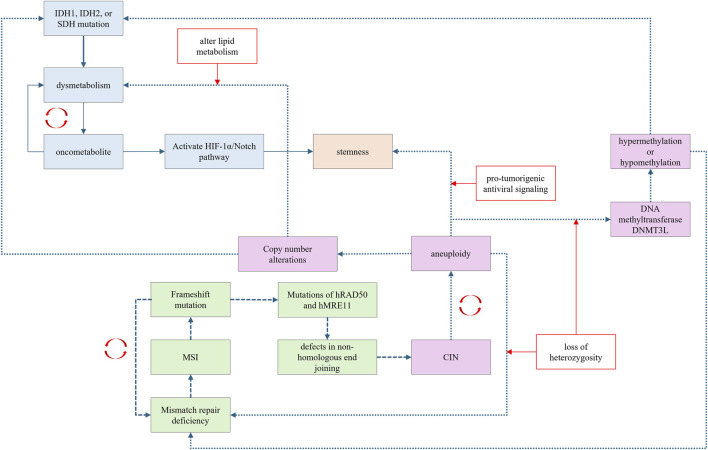
Carcinogenesis trajectories have extensive crosstalk and promote each other via distinctive mechanisms.

Intracellular signaling pathways exhibit extensive crosstalk, allowing a single oncogenic signal to initiate a cascade that activates a series of downstream oncogenic signals. While cancer may initially arise from a specific carcinogenesis trajectory, it can later involve mechanisms from other trajectories as the disease progresses (Figure 5). Trajectory shifts can occur during carcinogenesis, influenced by fluctuations in the underlying driving forces.

Recently, FH was demonstrated to function as a DNA repair factor in the non-homologous end-joining (NHEJ) process. Mutations in *FH* can impair the NHEJ process, potentially leading to CIN, thereby establishing a link between metabolic dysregulation and CIN. Furthermore, the accumulation of the oncometabolite fumarate can induce epithelial-mesenchymal transition, which is associated with the activation of stemness signaling (Sciacovelli et al., 2016). Oncometabolites can also activate the HIF-1α/Notch pathway, which is involved in cellular dedifferentiation and stemness maintenance (Xiang and Semenza, 2019; Mu et al., 2021).

Theoretically, in the chromosomal instability carcinogenesis trajectory, aneuploidy can disrupt mismatch repair systems through loss of heterozygosity, leading to MSI. Aneuploidy may also affect metabolism-related genes, leading to oncometabolite accumulation. For example, aneuploidy involving chromosome 21 can alter the dosage of genes encoding the DNA methyltransferase DNMT3L, causing hypermethylation or hypomethylation at CpG sites (Muskens et al., 2021; Lu et al., 2016). Aberrant methylation of CpG islands in gene promoters can inactivate tumor suppressor genes, thereby promoting carcinogenesis (Otani et al., 2013). CIN can also promote stemness by activating pro-tumorigenic antiviral signaling (Baba et al., 2024). Aneuploidy, such as the loss of chromosome arm 8p, can alter lipid metabolism and lead to dysmetabolism (Cai et al., 2016).

Frameshift mutations induced by MSI can affect genes responsible for chromosomal integrity (Koh et al., 2005). Both *hRAD50* and *hMRE11* are involved in the NHEJ repair process, and MSI-induced frameshift mutations in these genes contribute to CIN (Koh et al., 2005). Moreover, MSI-related frameshift mutations can involve non-coding regions, resulting in aberrant production of microRNAs (miRNAs). These dysregulated miRNA may further promote copy number amplifications or deletions.

In the stemness carcinogenesis trajectory, cells with stem-like properties are also susceptible CIN, leading to an aneuploidy phenotype (Gao et al., 2016).

## 7 The carcinogenesis trajectories and cancer origin theory

Various models have been proposed to explain the origin of cancer (Jassim et al., 2023). Theories of cancer origin typically include the somatic mutation (Bailey et al., 2018), stem cell (Tu et al., 2021), mutator phenotype (Loeb, 2001), atavistic model (Lineweaver et al., 2021; Bussey and Davies, 2021), and bioenergetic theory (Gyamfi et al., 2022; Brunner and Finley, 2023; Martínez-Reyes and Chandel, 2021; Pavlova et al., 2022). Each theory successfully aligns with certain scenarios but fails to explain oncogenesis in others. For instance, cancer stem cell theory proposes that cancer-initiating cells are derived from normal stem or progenitor cells (Lytle et al., 2018). However, cancer stem cells (CSCs) have yet to be identified in many types of cancers (Ishizawa et al., 2010). Furthermore, the prevailing view today is that cancer has a genetic component. Therefore, the accumulation of genetic mutations and epigenetic alterations, which regulate cell replication, cell division, cell metabolism, and cell growth, drive oncogenesis (Hanahan, 2022). However, there are reports that many carcinogens do not damage DNA (Riva et al., 2020), and simple oncogenic mutations are not sufficient for initiating tumorigenesis without additional regulatory factors, such as the microenvironment (Yuan et al., 2024). Emerging evidence indicates the regulatory roles of dysregulated metabolism in carcinogenesis (Gyamfi et al., 2022; Kong et al., 2024). The dysregulation of mitochondria or oncometabolites has been proposed to drive oncogenesis. However, in some cancers, only genetic abnormalities are found, without abnormal mitochondrial function (Kiebish and Seyfried, 2005; Seyfried, 2015). The atavistic theory suggests that cancer onset and progression represent a type of reversion from a multicellular to a unicellular phenotype (Lineweaver et al., 2021; Bussey and Davies, 2021). During the evolution of multicellular organisms, genetic programs inherited from the unicellular stage—such as those regulating “unlimited proliferation” and “autonomous migration”—are silenced.

In this context, the proposal of an integrated and rational theory on the origin of cancer is crucial. Here, we propose that carcinogenesis trajectories suggest that cancer may have multiple origins. Each trajectory can independently contribute to carcinogenesis; additionally, they may crosstalk and collectively promote the activation and accumulation of oncogenic signals. For instance, the mutator phenotype trajectory is aligned with somatic mutation, both of which focus on “mutations”. The dysmetabolic trajectory align with the bioenergetic theory, both of which emphasize the oncogenic effects of metabolic alterations. The stemness trajectory is aligned with cancer stem cells theory of cancer origin. Another interesting finding is that unicellular organisms lack the complex DNA repair mechanisms and cell cycle checkpoints found in multicellular organisms. They have a higher probability of chromosomal replication errors, fragment recombination, or loss, resulting in an inherently high rate of chromosomal variation. This is not a “defect” but a necessary adaptation for survival, as it enables them to rapidly generate genetic diversity to adapt to adverse environments. Based on this view, the CIN trajectory is partially aligned with atavistic theory.

## 8 Spontaneous-reinforcing oncogenic loop

The activation of oncogenes and the inactivation of tumor suppressor genes do not always lead to cancer, as pro-tumorigenic signals may be neutralized or buffered by redundant cellular signaling pathways. Therefore, the oncogenesis process depends on the cellular context. Based on the carcinogenesis trajectories discussed above, a notable finding emerges-for malignant transformation to happen, the initial oncogenic driver itself needs to self-reinforce, thereby promoting the progression of oncogenic events. For example, the degree of aneuploidy can spontaneously worsen owing to genomic instability induced by the aneuploidy itself (Burrell et al., 2013; Lamm et al., 2016; Ly et al., 2019; Garribba and Santaguida, 2022; Nicholson and Cimini, 2013). Oncometabolites generated through dysmetabolism can disrupt mitochondrial function, exacerbating metabolic dysfunction (Li et al., 2015). Moreover, the mutator phenotype resulting from mismatch repair deficiency can impair the function of other normal mismatch repair genes (Yamamoto and Imai, 2015), progressively accelerating the mutation rate. These findings suggest that the primary drivers of carcinogenesis should be specific oncogenic events which are capable of self-reinforcing, maintaining, and amplifying their effects. The formation of a spontaneous, self-reinforcing oncogenic loop may be necessary for the initiation of cancer.

## 9 Therapeutic significance of carcinogenesis trajectory theory

The origin of a tumor underlies a distinct tumorigenic pathway that directly affects its phenotype. A typical example is cancers originating from mismatch repair deficiency, which exhibit an MSI phenotype regardless of whether their origin is hereditary or sporadic (Imai and Yamamoto, 2008). Cancers of distinct origins generally respond differently to different treatments; therefore, targeting the carcinogenesis trajectory and halting the spontaneous-reinforcing oncogenic loop may be a potential treatment strategy. Various drugs have been designed and implemented in clinical practice to treat cancers by targeting specific hallmarks. One such hallmark is the production of oncometabolites, which occupy a central role in the “dysmetabolism–oncometabolites–dysmetabolism” spontaneous-reinforcing oncogenic loop. Therapeutic strategies have been designed to target the production of these oncometabolites. For example, the accumulation of the oncometabolite 2HD, which is caused by *IDH* mutations, can lead to oncogenesis (Gunn et al., 2023). A small-molecule inhibitor of mutant *IDH1*, which reduces the production of 2-HD, has been used to treat *IDH*-mutated acute myeloid leukemia (Mellinghoff et al., 2020) and cholangiocarcinoma (Abou-Alfa et al., 2020). The second hallmark involves the survival advantage of cells with stemness characteristics, which is essential to cancers originating from the stemness carcinogenesis trajectory. Various strategies have been designed to target cancer stemness. For example, all-trans retinoic acid has been designed to target stemness-related genes, such as *ALDH*, *SOX2*, and *KLF4*, to target cancer stem cells in gastric cancer (Nguyen et al., 2016). Tranylcypromine analogs have been used to induce cancer stem cell differentiation in the treatment of acute leukemia (Harris et al., 2012). A third targeted hallmark is aneuploidy, which can induce genomic instability and further exacerbate aneuploidy by itself. Therapeutic strategies targeting aneuploid cells have been designed for cancer treatment (Bhatia et al., 2024; Sdeor et al., 2024; Rendo et al., 2025). Another hallmark is DNA methylation, which can be caused by dysmetabolism. DNA methyltransferase inhibitors can reverse DNA hypermethylation, and hypomethylation at CpG regions enables the re-expression of silenced tumor suppressor genes in cancer cells (Lee et al., 2024). We hypothesize that the anti-tumor effects of these drugs may be attributed, at least in part, to their disruption of spontaneous-reinforcing oncogenic loops.

## 10 Conclusions and future perspectives

The origin of cancer could be fundamentally attributed to the aberrant activation of oncogenic signals. These pro-tumorigenic signals engaged in an extremely complex crosstalk, unlocking the plasticity of cancer cells and complicating cancer treatment. Although oncogenic signaling pathways often interact to varying degrees, there is still a predominant pathway by which cancer originates and acquires cancer-specific phenotypes and hallmarks; this pathway is defined as “the carcinogenesis trajectory”. In this article, we reviewed several typical carcinogenesis trajectories, including genomic instability trajectories (CIN and MSI), dysmetabolism-driven carcinogenesis trajectory, and stemness-related carcinogenesis trajectory, and discussed the crosstalk between them. The carcinogenesis trajectory is a dynamic process as well as a cellular state in which a specific phenotype can become self-sustaining and self-amplifying. Notably, a spontaneous-reinforcing carcinogenesis loop can be observed in each trajectory. This “carcinogenesis loop” possesses a self-reinforcing ability that progressively maintains and amplifies oncogenic signals, ultimately driving the emergence of cancer. Therefore, targeting the carcinogenesis trajectory and halting the carcinogenesis loop could serve as a potential strategy for cancer prevention and treatment.

## Data Availability

The original contributions presented in the study are included in the article/supplementary material, further inquiries can be directed to the corresponding author.
